# “Valve-In-Valve” Mitral por Via Transeptal: Um Papel de Destaque no Tratamento da Disfunção de Biopróteses no Brasil?

**DOI:** 10.36660/abc.20200575

**Published:** 2020-09-18

**Authors:** Dimytri Alexandre Alvim de Siqueira, Auristela Isabel de Oliveira Ramos, Fausto Feres

**Affiliations:** 1 Instituto Dante Pazzanese de Cardiologia São Paulo SP Brasil Instituto Dante Pazzanese de Cardiologia - Cardiologia Invasiva,São Paulo, SP – Brasil; 2 Instituto Dante Pazzanese de Cardiologia São Paulo SP Brasil Instituto Dante Pazzanese de Cardiologia – Valvopatias,São Paulo, SP – Brasil

**Keywords:** Valva Mitral/fisiopatologia, Substituição da Valva Aórtica Transcateter/tendências, Estenose da Valva Mitral, Biopróteses Valvulares Cardíacas, Diagnóstico por Imagem, Idoso

O tratamento percutâneo das valvopatias constitui tópico atual e de grande interesse na Cardiologia. A tecnologia inovadora e excelentes resultados clínicos obtidos com o TAVI revolucionaram a abordagem terapêutica da estenose aórtica do idoso e pavimentam o caminho para o tratamento transcateter das doenças da valva mitral e tricúspide. Sob esta perspectiva, o implante transcateter designado “*valve-in-valve*” tem sido empregado como uma opção terapêutica para pacientes de alto risco com disfunção de biopróteses cirúrgicas em posição aórtica.

Nesta edição dos Arquivos Brasileiros de Cardiologia, Nicz et al. relatam uma série de casos com 17 pacientes submetidos a procedimentos “valve-in-valve” em posição mitral, utilizando o acesso transeptal (TViVm) e empregando os dispositivos SAPIEN XT^®^ ou SAPIEN 3^®^ (Edwards Lifesciences)^1^ - originalmente criados e aplicados para o tratamento transcateter da estenose aórtica, mas recentemente aprovados para o tratamento da disfunção mitral bioprotética no Brasil. Os pacientes foram considerados de alto risco para reoperação (média de idade de 77 anos, média da Sociedade de Cirurgiões Torácicos STS 8,7%) e eram bastante sintomáticos - todos com classe NYHA > III. A mortalidade em trinta dias foi de 5,9%, e o procedimento resultou em redução no gradiente transvalvar médio (12 ± 3,8 para 5,3 ± 2,6 mmHg, p<0,001), ganho na área da prótese (1,1 ± 0,6 para 2,2 ± 0,4 cm2, p<0,001), sem regurgitação paravalvar ou central significativa. Após um seguimento médio de 161 dias, a maioria dos pacientes apresentou uma melhora nos sintomas (87,5% na classe NYHA < II), com uma mortalidade geral de 11,8%. Os autores forneceram uma excelente descrição da técnica necessária para um procedimento bem-sucedido e devem ser parabenizados pelos resultados obtidos, consistentes com os resultados clínicos de relatos anteriores que descrevem experiências iniciais. Neste cenário - no qual a Cardiologia assiste e aclama a rápida e bem-vinda evolução no tratamento percutâneo da doença valvar -, já seria o momento do TViVm lançar-se como protagonista no tratamento da disfunção de bioprótese mitral?

## Deterioração Estrutural, Falência de Biopróteses e Cirurgia de Retroca Valvar

As biopróteses são os dispositivos de escolha em pacientes idosos que necessitam de substituição valvar, sendo cada vez mais utilizadas também em indivíduos com menos de 65 anos e submetidos à cirurgia de troca valvar aórtica ou mitral.^2^ Embora estudos observacionais demonstrem uma excelente sobrevida-livre de complicações relacionadas às biopróteses,^3^ em última análise todas as próteses valvares biológicas estão propensas à deterioração, podendo tornar-se disfuncionantes. O termo deterioração estrutural valvar (SVD, do inglês *structural valve deterioration*) inclui alterações intrínsecas permanentes e irreversíveis da válvula (por ex., laceração no folheto, calcificação, formação de *pannus* ou fibrose); outras causas patológicas da disfunção da válvula bioprotética (por ex., trombose, endocardite) são potencialmente reversíveis. Quando ocorre comprometimento hemodinâmico significativo (regurgitação grave ou estenose) e/ou há um evento clínico relacionado à SVD (reintervenção, morte), o termo insuficiência valvar bioprotética (BVF, *bioprosthetic valve failure*) é atualmente sugerido.^4^

Em todo o mundo, a cirurgia de retroca valvar é indicada para a maioria dos pacientes com SVD. Contudo, o risco de uma reoperação valvar mitral (rTVM) permanece maior do que o risco de uma primeira cirurgia. Idade avançada, sexo feminino, classe NYHA pré-operatória, fração de ejeção do ventrículo esquerdo (FEVE) reduzida, hipertensão pulmonar e número de cirurgias anteriores foram todos identificados como preditores de mortalidade. Além disso, as aderências de cirurgias anteriores aumentam substancialmente as complicações hemorrágicas. Em um estudo com 260 pacientes inscritos no *European Redo Cardiac Operation Database* (RECORD), a mortalidade hospitalar foi de 9,2% após a rTVM. Neste registro, foi relatada uma alta incidência de complicações pós-operatórias, como síndrome de baixo débito cardíaco (17,3%), necessidade de suporte circulatório (9,2%), insuficiência renal aguda (16,5%) e necessidade de transfusão (25%).^5^ Da mesma forma, em registro contemporâneo da *Society of Thoracic Surgery* (STS) com 11.973 pacientes revelou que a rrTVM (n = 1096) esteve associada a uma maior mortalidade (11,1%) quando comparada à primeira cirurgia de troca ou plastia (6,5%).^6^ Apesar da escassez de dados sobre rTVM no Brasil, sabemos que a cardiopatia reumática com envolvimento valvar constitui frequente indicação para cirurgia em pacientes jovens. Como a ocorrência de BVF é influenciada pela idade – em indivíduos com menos de 40 anos, por exemplo, a taxa de reoperação após 15 anos pode ser de 64%^7^ –, muitos de nossos pacientes necessitam múltiplas cirurgias com riscos crescentes, impacto substancial na qualidade de vida e custos para o sistema de saúde.

## Resultados Clínicos e Ecocardiográficos Após TVIVm e Benefícios Potenciais do Acesso Transeptal

O *valve-in-valve* mitral tem sido adotado como uma alternativa menos invasiva de tratamento para a SVD em indivíduos idosos e/ou alto risco cirúrgico. O procedimento pode ser realizado através de abordagens cirúrgicas ou percutâneas. A abordagem cirúrgica transcateter inclui o acesso transapical ou atrial esquerdo direto (por toracotomia). Como destacado por Nicz et al., uma abordagem totalmente percutânea pode ser alcançada com segurança através da veia femoral (ou jugular) com punção transeptal. Apesar da falta de dados conclusivos, o acesso transeptal tem sido associado a vantagens clínicas em alguns estudos. No registro VIVID, os pacientes que tinham FEVE basal reduzida e foram tratados via acesso transeptal apresentaram melhor recuperação da função ventricular esquerda do que os pacientes submetidos ao TViVm por acesso transapical – possivelmente relacionado a lesão miocárdica decorrente deste tipo de abordagem cirúrgica.^8^ Recentemente apresentada por Guerrero et al.,^9^ uma análise do registro STS/ACC TVT (n = 1.576) mostrou que, nos EUA, a maioria dos procedimentos (84,1%) efoi realizada por acesso transeptal: embora a a taxa de sucesso técnico não tenha sido diferente entre os grupos (transeptal 97,1%, transapical 94,6%), a utilização da via transeptal esteve associada a uma menor mortalidade cardiovascular em 30 dias (1,8% vs. 4,4%; p = 0,03), com redução do tempo de internação hospitalar (2 vs. 6 dias; p <0,001). A mortalidade por todas as causas em 1 ano também foi menor no grupo transeptal (15,8% *vs*. 21,7%; HR 0,67; IC 95% 0,47-0,97).^9^

A punção transeptal, guiada pela ecocardiografia, constitui a etapa fundamental da técnica percutânea e requer expertise. No registro STS/ACC TVT (n = 680),^10^ as complicações potenciais maiores relacionadas ao procedimento incluíram perfuração cardíaca (1,9%), tamponamento e conversão para cirurgia cardíaca (1,3%). A necessidade de fechamento da comunicação interatrial residual foi baixa (5,4%) e acidente vascular cerebral ou ataque isquêmico transitório ocorreu em 1,6%. A obstrução da via de saída do ventrículo esquerdo (VSVE) é uma complicação aguda temida da TViVm, e a alta incidência (11,7%) descrita por Nicz e cols. pode ser justificada pelo pequeno tamanho da amostra e/ou à curva de aprendizado na seleção de pacientes. Com efeito, a tomografia computadorizada (TC) multi-slice é de suma importância para se detectar características de alto risco para obstrução da VSVE; em pacientes adequadamente selecionados para TViVm, a incidência de obstrução da VSVE é menor (0,7%) do que a observada em implantes após anuloplastia, denominados “valve-in-ring” (4,9%) ou em situações de calcificação do anel com “valve-in-MAC” (10%).^11,12^ A alcoolização septal^11^ pré-procedimento ou a técnica modificada de laceração transcateter do folheto da bioprótese (LAMPOON) podem ser indicadas para pacientes com risco de obstrução da VSVE durante o TMViV.^12^

Em comparação ao “*valve-in-valve*” aórtico, a ocorrência de gradientes residuais mais elevados após o TViVm é incomum, pois geralmente as biopróteses cirúrgicas na posição mitral são de maiores dimensões (acima de 29 mm). Na presente coorte, todas as próteses cirúrgicas que apresentavam disfunção eram > 27 mm, e os baixos gradientes transprotéticos pós-procedimento persistiram no seguimento de 30 dias. Assim, a indicação de TViVm deve ser cuidadosamente avaliada em pacientes com biopróteses menores (especialmente aqueles com estenose): novas técnicas, como a fratura da válvula cirúrgica bioprotética com balões não complacentes durante o TMViV, podem ser úteis nesse cenário.^13^

## As Questões não Respondidas

Embora seja um procedimento menos invasivo, seguro e com eficácia demonstrada, diversas incertezas associadas ao TViVm devem ser enfatizadas.

A consolidada experiência cirúrgica revela que a trombose valvar é mais frequente na posição mitral (*vs.* aórtica), e isso também pode ser semelhante ao se utilizar válvulas cardíacas transcateter. No presente estudo, não foram detectados casos de trombose valvar, mas o seguimento clínico médio foi muito curto (menos de 6 meses) e a avaliação ecocardiográfica foi limitada a 30 dias. Os autores não relatam o tipo de terapia antiplaquetária ou anticoagulante empregada após o TViVm, e na atualidade não há padronização dos regimes antitrombóticos prescritos após o procedimento. Em um registro multicêntrico, ocorreu trombose da válvula cardíaca transcateter (THV, do inglês *transcatheter heart valve*) em 10 casos (9 TMViV e 1 *valve-in-ring*), e a taxa acumulada de 1 ano de trombose da THV foi significativamente maior nos pacientes sem anticoagulação, em comparação com aqueles anticoagulados (6,6% *vs.* 1,6%; p = 0,019).^14^ Assim, preconiza-se que uma avaliação ecocardiográfica deva ser realizadas periodicamente e - caso observado elevação nos gradientes transprotéticos - , a presença de trombose deve ser excluída idealmente com tomografia computadorizada de alta resolução.

A insuficiência tricúspide funcional constitui fator de mau prognóstico e está intimamente relacionada à manutenção de sintomas e reinternações em pacientes com doença valvar mitral. De fato, diretrizes preconizam o reparo cirúrgico da válvula tricúspide concomitante à cirurgia de troca valver mitral.^15^ Uma vez que a insuficiência tricúspide, hipertensão pulmonar, dilatação e insuficiência do ventrículo direito (VD) geralmente estão presentes em pacientes com BVF mitral, os efeitos do TViVm sobre a válvula tricúspide e a hemodinâmica do VD também devem ser investigados. A redução da pressão arterial sistólica pulmonar, descrita por Nicz et al., é encorajadora, mas nenhuma informação sobre regurgitação da válvula tricúspide foi fornecida: praticamente, 25% dos pacientes nessa coorte foram re-hospitalizados e 1 paciente morreu de insuficiência cardíaca congestiva. Tais aspectos relacionados às câmaras direitas devem ser considerados para a tomada de decisão em pacientes adequados tanto para rTVM como para TViVm.

Finalmente, embora a durabilidade de uma prótese transcateter em posição mitral possa não ser uma questão tão relevante em indivíduos idosos e/ou inoperáveis, o conhecimento a respeito de quão durável é o procedimento torna-se essencial para justificar e orientar as recomendações clínicas para pacientes mais jovens com BVF – como, por exemplo, aqueles portadores de doença reumática. Sob esta perspectiva, o estudo nacional, prospectivo e randomizado SURViV (NCT04402931) tem como objetivo avaliar os resultados clínicos a curto e longo prazos da TViVm, comparativamente à rTVM. Em futuro próximo, seus resultados podem trazer contribuições para se definir o papel do valve-in-valve mitral em nosso meio.
