# Supplementary material for: “Valve-In-Valve” Mitral por Via Transeptal: Um Papel de Destaque no Tratamento da Disfunção de Biopróteses no Brasil?
Source: Arq Bras Cardiol. 2020 Sep 18;115(3):525–7. [Article in Portuguese] doi: 10.36660/abc.20200575 (PMC9363078; doi:10.36660/abc.20200575)
Supplement: Translation

# Translation: Transseptal, Transcatheter Mitral Valve-In-Valve Replacement: Ready for Prime Time Treatment of Bioprosthetic Valve Failure in Brazil?

**Authors:** Dimytri Alexandre Alvim de Siqueira, Auristela Isabel de Oliveira Ramos, Fausto Feres

**Affiliations:** 1 Brazil Instituto Dante Pazzanese de Cardiologia - Cardiologia Invasiva, São Paulo, SP - Brazil; 2 Brazil Instituto Dante Pazzanese de Cardiologia – Valvopatias, São Paulo, SP – Brazil

**Keywords:** Mitral Valve/physiopathology, Transcatheter Aortic Replacement/trends, Mitral Valve Stenosis, Heart Valve Prosthesis, Diagnosis, Imaging, Aged

Less-invasive, transcatheter-based treatment of valvular heart disease is a topic of major interest in cardiology nowadays. The breakthrough technology and unprecedented clinical results of transcatheter aortic valve replacement revolutionized the therapeutic approach for aortic stenosis and paved the way for percutaneous treatment of mitral and tricuspid disease. In this regard, transcatheter mitral valve-in-valve implantation (TMViV) has emerged as a feasible option for patients with surgical bioprosthetic valve dysfunction.

In this issue of *Arquivos Brasileiros de Cardiologia*, Nicz et al. report a case series of 17 patients who underwent transseptal TMViV using SAPIEN XT^**®**^ or SAPIEN 3^**®**^ (Edwards Lifesciences) devices^1^ - originally created and applied for transcatheter treatment of aortic stenosis but recently approved for the treatment of mitral bioprosthetic dysfunction in Brazil. Patients were considered high-risk for reoperation (mean age 77 years, mean Society of Thoracic Surgeons STS 8.7%) and were very symptomatic - all in NYHA class > III. Thirty-day mortality was 5.9%, and a reduction in the mean transvalvular gradient (12 ± 3.8 to 5.3 ± 2.6 mmHg, p<0.001) with an increase in the bioprosthetic area (1.1 ± 0.6 to 2.2 ± 0.4 cm^2^, p<0.001) were observed, without significant paravalvular or central regurgitation. After a mean follow-up of 161 days, the majority of patients experienced an improvement in symptoms (87.5% in NYHA class < II) with an overall mortality of 11.8%. The authors provided an excellent description of the technique required for a successful procedure and should be congratulated for the results obtained, consistent with the clinical outcomes from previous reports that described initial experiences. As cardiology beholds the fast-evolving field of percutaneous treatment of valvular heart disease, is it time for TMViV to be adopted as an alternative therapy for patients with mitral bioprosthetic valve dysfunction?

## Structural Bioprosthetic Valve Deterioration, Failure and Redo Valve Surgery

Bioprosthetic valves are the device of choice for older patients who require left heart valve replacement, and recent data indicate that bioprostheses are also increasingly used in patients less than 65 years of age who undergo aortic or mitral valve surgeries.^2^ Although numerous observational studies have shown excellent survival and freedom from valve-related complications,^3^ ultimately all bioprosthetic heart valves are prone to fail. The term structural valve deterioration (SVD) includes permanent, irreversible intrinsic changes of the valve (i.e. leaflet tears, calcification, pannus formation or fibrosis); other pathological causes of bioprosthetic valve dysfunction (i.e. thrombosis, endocarditis) are potentially reversible. When significant hemodynamic compromise (severe regurgitation or stenosis) ensues and/or there is a clinical event related to SVD (reintervention, death), the term bioprosthetic valve failure (BVF) is now suggested.^4^

Redo surgical valve replacement is currently indicated for the majority of patients with bioprosthetic SVD. The risk of redo mitral surgery remains much higher than the risk of first mitral valve replacement (MVR). Advanced age, female gender, preoperative NYHA class, reduced left ventricular ejection fraction (LVEF), pulmonary hypertension and the number of prior operations have all been identified as predictors of mortality. Moreover, adhesions from previous surgery substantially increases bleeding complications. In a study with 260 patients enrolled in the European Redo Cardiac Operation Database (RECORD), in-hospital mortality was 9.2% after redo MVR. A high incidence of postoperative complications, such as low cardiac output syndrome (17.3%), need for circulatory support (9.2%), acute renal failure (16.5%) and need for transfusion (25%) was reported.^5^ Similarly, a contemporary study from the Society of Thoracic Surgery (STS) with 11,973 patients revealed that mitral reoperation (n=1096) was associated with higher operative mortality (11.1%) in comparison with the first MVR or repair (6.5%).^6^ Despite the paucity of data about redo MVR in Brazil, it is well-known that rheumatic heart disease with valvular involvement is the most frequent indication for cardiac valve surgery in young patients. Since BVF is influenced by age – in patients less than 40 years of age, for example, 15-year freedom from reoperation can be as low as 36%^7^-, patients in our country require multiple reinterventions with increasing risks, substantial impact on their quality of life and costs to the healthcare system.

## Clinical and Echocardiographic Outcomes After TMViV And Potential Benefits of a Less Invasive, Transseptal Approach

TMViV has been adopted in many centers worldwide as a less invasive alternative to redo MVR in older individuals at high surgical risk. The procedure can be performed through surgical or percutaneous approaches. The surgical transcatheter approach includes the transapical or direct left atrial (by thoracotomy) access. As highlighted by Nicz et al.,^8^ a fully percutaneous approach can be safely achieved through the femoral (or jugular) vein with transseptal puncture. Although conclusive data are lacking, the transseptal access has been associated with clinical advantages in some studies. In the VIVID registry, patients who had reduced LVEF at baseline and were treated via transseptal access had better recovery of left ventricular function than patients who underwent TMViV by transapical access – possibly related to myocardial injury associated with the surgical approach.^8^ Recently presented by Guerrero et al.,^9^ an analysis from the STS/ACC TVT Registry (n=1576) showed that in the US, most procedures (84.1%) were now performed using transseptal access: although technical success was not statistically different between the groups (transseptal 97.1%, transapical 94.6%), transseptal access was associated with a lower rate of cardiovascular death at 30 days (1.8% vs 4.4%; p = 0.03), with a reduced median hospital length of stay (2 vs. 6 days; p < 0.001). All-cause mortality at 1 year was also lower in the transseptal group (15.8% vs 21.7%; HR 0.67; 95% CI 0.47-0.97).^9^

Echocardiographic-guided, transseptal puncture is the fundamental step of percutaneous TMViV, and requires expertise. In the STS/ ACC TVT registry (n=680),^10^ potential major procedural complications included cardiac perforation (1.9%), tamponade and conversion to open-heart surgery (1.3%). Need for closure of the residual atrial septal defect was low (5.4%) and stroke or transient ischemic attack occurred in 1.6%. Left ventricular outflow tract (LVOT) obstruction is a feared acute complication of transcatheter MVR, and the high incidence (11.7%) described in the present cohort may be due to the small sample size and selection bias. Pre-procedural planning using multi-slice computed tomography (CT) is of paramount importance to detect high-risk features for LVOT obstruction and, in properly selected TMViV patients, the incidence of LVOT obstruction in larger studies is lower (0.7%) than in transcatheter mitral valve-in-ring (4.9%) or valve-in-mitral annular calcification (10%).^11,12^ Pre-procedure alcohol septal ablation^11^ or the modified technique of transcatheter laceration of the bioprosthetic leaflet (LAMPOON) may be indicated in patients at risk for LVOT obstruction during TMViV.^12^

As compared to transcatheter aortic valve-in-valve procedures, the occurrence of high residual gradients after TMViV is uncommon, since larger surgical bioprostheses are usually implanted in the mitral position. In the present cohort, all labeled surgical valves were > 27 mm, and post-procedure mean transvalvular gradients were low and sustained at 30 days. Indeed, TMViV should be carefully considered in patients with smaller bioprosthetic valves (especially those with stenosis): new techniques such as bioprosthetic surgical valve fracture with non-compliant balloons during TMViV may be helpful in this scenario.^13^

## The Unresolved Issues

Although it is a less invasive procedure with good safety and efficacy results, several unresolved issues and uncertainties associated with TMViV should be emphasized.

Experience from conventional valve surgery suggests that valve thrombosis is more frequent in the mitral (vs. aortic) position, and this could be also true for transcatheter heart valves. In the present study, no cases of valve thrombosis were detected but mean clinical follow-up was too short (less than 6 months) and echocardiographic assessment was limited to 30 days. Antiplatelet or anticoagulation therapy after TMViV was not mentioned by the authors, and in reality there is a lack of standardization of antithrombotic regimens prescribed after transcatheter valve therapies. In a multicenter registry, THV thrombosis occurred in 10 cases (9 TMViV and 1 valve-in-ring), and the cumulative 1-year rate of THV thrombosis was significantly higher in patients without anticoagulation, as compared to those with anticoagulation (6.6% vs. 1.6%; p = 0.019).^14^ Accordingly, systematic echocardiographic evaluations should be performed periodically, and if an increase in transmitral gradients is observed, the presence of thrombosis has to be excluded, ideally with high-resolution CT imaging.

Functional, tricuspid regurgitation has a negative prognostic effect on survival and is closely related to symptoms and re-hospitalizations in patients with mitral disease. Accordingly, concomitant surgical tricuspid repair is currently recommended at the time of left-heart valve surgery.^15^ As tricuspid insufficiency, pulmonary hypertension, right ventricular (RV) dilation and failure are usually present in patients with mitral BVF, the effects of TMViV on tricuspid and RV hemodynamics should be also investigated. The reduction in pulmonary systolic arterial pressure described by Nicz et al. is encouraging, but no information regarding tricuspid regurgitation was provided: indeed, 25% of patients in their cohort were re-hospitalized, and 1 patient died of congestive heart failure. Those aspects should be considered for clinical decision making in suitable patients for both redo MVR and TMViV.

Finally, learning about transcatheter mitral valve durability may not be a relevant issue in older, inoperable patients but it is essential to justify and guide clinical recommendations for younger surgical candidates with BVF – like those with rheumatic heart disease. Answers to all these questions are difficult to obtain from non-randomized, non-controlled trials. In this regard, the SURViV trial (NCT04402931) - a randomized trial comparing TMViV and redo MVR - is currently enrolling patients in our country and will shed more light on this new procedure.
